# DC-ATLAS: a systems biology resource to dissect receptor specific signal transduction in dendritic cells

**DOI:** 10.1186/1745-7580-6-10

**Published:** 2010-11-19

**Authors:** Duccio Cavalieri, Damariz Rivero, Luca Beltrame, Sonja I Buschow, Enrica Calura, Lisa Rizzetto, Sandra Gessani, Maria C Gauzzi, Walter Reith, Andreas Baur, Roberto Bonaiuti, Marco Brandizi, Carlotta De Filippo, Ugo D'Oro, Sorin Draghici, Isabelle Dunand-Sauthier, Evelina Gatti, Francesca Granucci, Michaela Gündel, Matthijs Kramer, Mirela Kuka, Arpad Lanyi, Cornelis JM Melief, Nadine van Montfoort, Renato Ostuni, Philippe Pierre, Razvan Popovici, Eva Rajnavolgyi, Stephan Schierer, Gerold Schuler, Vassili Soumelis, Andrea Splendiani, Irene Stefanini, Maria G Torcia, Ivan Zanoni, Raphael Zollinger, Carl G Figdor, Jonathan M Austyn

**Affiliations:** 1 Department of Pharmacology, University of Firenze, Firenze, Italy; 2 Department of Tumor Immunology, NCMLS, Radboud University Nijmegen Medical Centre, Nijmegen, The Netherlands; 3 Department of Biology, University of Padua, Padova, Italy; 4 Department of Cell Biology and Neurosciences, Istituto Superiore di Sanità, Roma, Italy; 5 Department of Pathology and Immunology, Faculty of Medicine, University of Geneva, Geneva, Switzerland; 6 Department of Dermatology, University of Erlangen, Erlangen, Germany; 7 Leaf Bioscience, Milano, Italy; 8 Novartis Vaccines, Siena, Italy; 9 Department of Computer Science, Wayne State University, Michigan, USA; 10 Marseille-Luminy Immunology Center, Université de la Méditerranée, Marseille, France; 11 Department of Biotechnology and Biosciences, University of Milano-Bicocca, Milano, Italy; 12 Department of Gastroenterology, Radboud University Nijmegen Medical Centre, Nijmegen, The Netherlands; 13 Institute of Immunology, University of Debrecen, Debrecen, Hungary; 14 Department of Immunohematology and Bloodtransfusion, Leiden University Medical Center, Leiden, The Netherlands; 15 Miravtech Corporation, Michigan, USA; 16 Department of Immunology, Institute Curie, Paris, France; 17 Department of Clinic Physiopathology, University of Firenze, Firenze, Italy; 18 Nuffield Department of Surgery, University of Oxford, Oxford, UK

## Abstract

**Background:**

The advent of Systems Biology has been accompanied by the blooming of pathway databases. Currently pathways are defined generically with respect to the organ or cell type where a reaction takes place. The cell type specificity of the reactions is the foundation of immunological research, and capturing this specificity is of paramount importance when using pathway-based analyses to decipher complex immunological datasets. Here, we present DC-ATLAS, a novel and versatile resource for the interpretation of high-throughput data generated perturbing the signaling network of dendritic cells (DCs).

**Results:**

Pathways are annotated using a novel data model, the Biological Connection Markup Language (BCML), a SBGN-compliant data format developed to store the large amount of information collected. The application of DC-ATLAS to pathway-based analysis of the transcriptional program of DCs stimulated with agonists of the toll-like receptor family allows an integrated description of the flow of information from the cellular sensors to the functional outcome, capturing the temporal series of activation events by grouping sets of reactions that occur at different time points in well-defined functional modules.

**Conclusions:**

The initiative significantly improves our understanding of DC biology and regulatory networks. Developing a systems biology approach for immune system holds the promise of translating knowledge on the immune system into more successful immunotherapy strategies.

## Background

Dendritic cells (DCs) orchestrate a repertoire of immune responses that endow resistance to infections and tolerance to self. DC plasticity has a prominent role in eliciting the proper immune response. Different DC subsets display different receptors and surface molecules and express different sets of cytokines/chemokines, all of which lead to distinct immunological outcomes. Among the receptors are the innate pattern recognition receptors (PRRs) that mediate the initial sensing of an infection. These include Toll-like receptors (TLRs), RIG-I-like receptors (RLRs), NOD-like receptors (NLRs), and C-type lectin receptors (CLRs) [1]. TLRs recognize conserved structures of microbes and are localized on the cell surface (TLR1, TLR2, TLR4, TLR5 and TLR6) to recognize bacterial and fungal cell wall components or in intracellular membranes such as endosomes or phagosomes (TLR3, TLR7, TLR8 and TLR9) where they recognize viral or microbial nucleic acids [1]. Thus, different TLRs are amenable to targeting by different types of agents [2].

Because of their essential role in the initiation of an adaptive immune response, DCs are an attractive target for therapeutic manipulation of the immune system [3]. In fact, DC physiology is one of the research areas where basic knowledge has been more readily translated into clinical applications. DC-based vaccines have been rapidly transferred from the laboratory to the clinic. However, it is evident that, after more than ten years of worldwide experience with DC vaccination, the therapeutic potential of these cells has not yet been entirely exploited [4]. We thus need to improve our understanding of the complex biology of these cells [5] that operate at the crossroad of innate and adaptive immunity. The complexity and heterogeneity of the DC system however, may require a shift from reductionism to more holistic systems biology approaches. We expect that more detailed insight in the signaling pathways that operate in DCs will open new perspectives for a better exploitation of their therapeutic potential.

Immune systems biology is defined as the comprehensive and quantitative study of interactions between hosts and microbes over time, leading to the generation of models describing their dynamic behavior of immune cells and pathogens.

Many studies investigated immune cell since these cells are particularly suited to functional genomics analyses because their responses to specific stimuli in a controlled environment can be clearly categorized. Innate responses against pathogens however cannot be considered as a set of discrete signaling pathways activated by a pathogen binding to a receptor; but rather such responses are composed of many interconnected pathways depending on multiple factors.

Important initiatives based on systems biology are arising to collect high throughput data and to develop sophisticated bioinformatic methods to compare and analyze these data. In this respect, the Immunological Genome Project initiative [6] represents the first transcriptomic project to apply a truly systems-level approach to the analysis of immune cell populations. Current publicly available pathway databases provide generic rather than thematic or cell-type specific pathways. Nevertheless, certain initiatives are proposing the cellular specificity of certain reactions. In recent studies [7] a comprehensive map of macrophage molecular interactions was created, including ligands such as PAMPs and interleukins as input signals, and the release of cytokines and lipids as output signals. Recently a macrophage specific pathways database valuable for computational modeling and for the interpretation of functional genomics data has been published [8]. At the time of writing, initiatives aiming at a better description of the signaling networks of DCs are underway [9].

Here we describe DC-ATLAS, a collection of pathways specifically curated in DC, that can be exploited, using pathway analysis based approach, in deciphering the complex network of interactions occurring in DCs upon activation. The pathways are available at http://www.dc-atlas.net and they cover a plethora of cell surface receptors (eg. TLRs, CLRs, NLRs) and DC-relevant processes (e.g antigen presentation, migration). To illustrate the potential of this new resource, we have selected as paradigmatic the set of TLRs pathways. We describe how they were curated and show the advantages of our approach through their validation both *"in silico" *and *"in vitro"*.

The database contains both human and mice data and the modular structure of DC-ATLAS led to unravel of the major differences between these two systems. The knowledge provided by DC-ATLAS permits the conversion of genomic research into accurate and robust biological hypotheses by generating signatures that serve as valuable tools to understand DC physiology and contribute to the design of new strategies in immunotherapy.

## Results

### Dendritic cells specific pathways in DC-ATLAS

DC-ATLAS is one of the first immunological and bioinformatics integrated project which complies with the Systems Biology Graphical Notation (SBGN) [10]. It is composed of a database holding signal transduction pathways extensively curated specifically for DCs.

Every specific gene and reaction were annotated providing information on the organism, the organism part, the cell type and the experimental details in which the evidence has been obtained. The community of curators within DC-ATLAS manually annotated the pathways providing also the most updated reference available in existing databases and literature, as well as generating experimental proofs in their own laboratories where these were lacking. The curation procedure itself is described in more detail in Additional file 1.

### Development of a specific data format for DC-ATLAS

To ensure that the results of the curation process would also be fully used for representation and data analysis, a DC specific data format, the Biological Connection Markup Language (BCML), was developed to represent pathways according to the specification proposed by the Systems Biology Graphical Notation (SBGN)[10]. BCML provides a machine-readable representation of the pathways, which can be used for description, manipulation, analysis and graphical representation. BCML is a format developed using XML and defines the complete Process Description (PD) specification from SBGN, including not only the definition of the elements, but also the rules and constraints needed to assemble a network.

In addition to a full implementation of the PD specification, BCML provides a series of optional features. First of all, BCML can include additional information on the entities that compose the network: each entity can be described by a series of species specific database identifiers, e.g. Entrez Gene or Uniprot accession numbers. Furthermore, each entity or reaction can have a set of facts or "Findings" associated. "Findings" are collections of biological information that are relevant to that entity or reaction. The current specification includes support for organism, organism part (tissue), cell type, the specific biological environment in which the evidence was proven, and the type of the experiment used to gather evidence. To reduce ambiguity and promote consistency among different "findings", the schema enforces a controlled vocabulary built from current medical ontologies.

The specification of BCML is accompanied by a series of programs (BCML software suite) that enable the use and manipulation of the format both for the bioinformatician and the biologist. First of all, the software suite permits validation of pathways described using BCML, to ensure consistency and the proper enforcement of the SBGN rules. Secondly, the software can create a fully SBGN compliant graphical representations by transforming the BCML XML into other formats (GraphML) which can be then saved as images with third-party software.

The format also permits filtering of the pathway data creating a new network containing only elements with user-defined characteristics, allowing the production of tailored made pathways, allowing individualized analyses. The tools in the BCML software suite allow specific "filtering" of the pathway, taking advantage of all the information stored. For example, nodes and edges can be selected for a specific cell type or organism, permitting the construction of customized network maps to represent specific biological contexts. When a filter is applied to the pathway, elements are marked as "included", "excluded", or "affected". An element of the pathway is included or excluded in the resulting map if it matches with the selected filter criteria or not. The "affected" state is used to indicate elements that may not be present depending on the filtering; for example. in a specific cell type a complex may not form if one or more of its proteins are not present. Filtering may be used to assist data analysis and interpretation and might point to gaps in current knowledge.

The BCML format can incorporate any kind of experimental measurements that can be matched to the identifiers of an element. This allows modification of the BCML map, facilitating incorporation of high-throughput data coming from transcriptomic or proteomic experiments. The outcome will be visualized in different color on the graphical map.

Finally, BCML allows transformation of the pathways into different data formats, which may be needed for further analysis. Tools provided within the suite allow the generation of identifier (gene) lists from a BCML file, enabling their use with analysis tools such as Gene Set Enrichment Analysis (GSEA), Fisher's Exact Test. Additionally, the format can be converted to a form amenable for impact analysis through the SPIA R package. This conversion can take into account the filtering applied to the elements of the pathway, to carry out individualized analyses.

A detailed description of BCML format is available as Additional file 2.

### TLR pathway curation and modular structure in DC-ATLAS

At present, the human TLR pathway set in DC-ATLAS is a network organized in an ensemble of 8 pathways (TLR1-2, TLR2-6, TLR3, TLR4, TLR5, TLR7, TLR8 and TLR9), subdivided in 10 sensing modules, 32 signal transduction modules and 30 outcome modules. In contrast to what is present in existing databases, TLR7 and TLR8 were curated separately. Although their genes lie in close proximity on chromosome X and are highly homologous, recent evidences suggest they have distinct roles in DC mediated immune response [11-13]. For example, despite the fact that both TLRs bind the same ligand and largely overlap in their signaling, stimulated TLR7 activates transcription factor IRF7 [14] while IRF1 [15] is only an effector of TLR8 mediated signaling.

**Figure 1:**
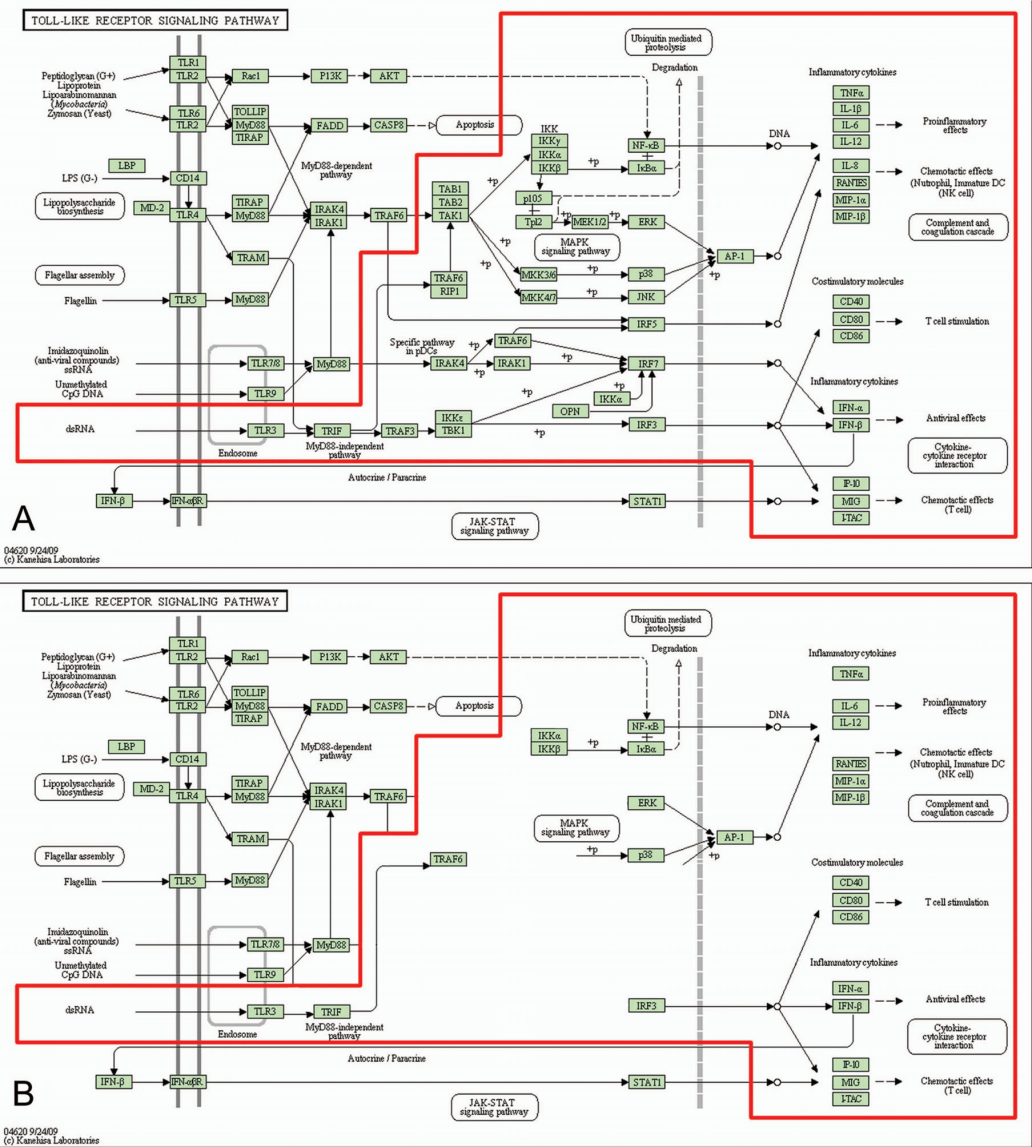
**Comparison of the DC-ATLAS Toll Like Receptor (TLR) 3 pathway with other pathway databases**. (A) Representation of the KEGG Toll-like receptor (TLR) pathway. The TLR3 signal is highlighted in red. (B) Representation of the KEGG TLR3 pathway, displaying only the reactions proven in human DCs. The curation led to the validation of about 50% of the genes previously belonging to public TLR3 signaling.

Expert-guided, manual curation of the pathways has been a crucial part of the DC-ATLAS initiative, leading to a substantial "reshaping" of the existing pathways. For example, curation of TLR3 pathway led to the validation of only about 50% of the genes included in the list originally retrieved from public databases (Figure 1A and 1B). Furthermore, a number of genes previously not annotated as belonging to the TLR3 pathway in publicly available databases were found to participate to the signaling cascade in DCs. Among them, especially the number of target genes has been substantially extended, including the cytokines IL-10 [16-19], IL-1α [17], the chemokines CCL3 [17] and the CCR7 chemokine receptor [20], the co-stimulatory molecule CD83 [16,20], the transcription factor STAT4 [21] and the enzyme INDO [19,22].

Another example demonstrating the importance of DC-ATLAS curation is exemplified by the fact that in the sensing module of TLR9 we found a new element, UNC93B1, whose involvement in signaling was demonstrated already in 2007 [23]. All the other improvements of DC-ATLAS with respect to existing pathways fall mainly in the signal transduction and outcome modules.

A summary of all the new genes and/or connections, not previously annotated in TLR pathways, and present in DC-ATLAS is presented in Table 1. Since the field is rapidly evolving, when new evidence appears demonstrating that new or so far excluded interactions are operating in DCs, DC-ATLAS will be updated accordingly.

**Table 1:** DC-ATLAS curation results: number and names of new genes present in TLR pathways of DC

TLR signaling pathways Cascade	Genes in revised pathway	New genes in revised pathway	Names of new genes or chemicals previously absent from the DCs pathway (Entrez Gene ID)
TLR1/TLR2	72	21	**Transduction modules: SOCS1 **(8651), **BTK **(695), **PPARG **(5468). **Outcomes: CXCL2 **(2920), **IL2 **(3558), **MAP3K8 **(1326), **MHC **(HLA-E (3133), HLA-DMB (3109), HLA-DOA (3111), HLA-DPA1 (3113), HLA-DPB1 (3115), HLA-DQA1 (3117), HLA-DQB1 (3119), HLA-DRA (3122), HLA-DRB1 (3123), HLA-DRB3 (3125), HLA-DRB4 (3126), HLA-DRB5 (3127)), **CD86 **(942), **CCL19 **(6363), **CCL2 **(6347).

TLR2/TLR6	78	26	**Transduction modules**: **SOCS1 **(8651), **BTK **(695), **PPARG**(5468), **PRKCA **(5578) **Outcomes**: **CXCL2 **(2920), **IL2 **(3558), **MAP3K8 **(1326), **MHC **(HLA-E (3133), HLA-DMB (3109), HLA-DOA (3111), HLA-DPA1 (3113), HLA-DPB1 (3115), HLA-DQA1 (3117), HLA-DQB1 (3119), HLA-DRA (3122), HLA-DRB1 (3123), HLA-DRB3 (3125), HLA-DRB4 (3126), HLA-DRB5 (3127)), **CD86 **(942), **CCL19 **(6363), **CCL2 **(6347), **IL10 **(3586), **CD80 **(941), **CD40 **(958), **CCR7 **(1236).

TLR3	66	23	**Transduction modules: PRKCA **(5578), **PRKCB1**(5579), **CREBBP **(1387), **SRC **(6714), **AKT1 **(207). **Outcomes: IL10 **(3586), **IL29 **(282618), **IL28A**(282616), **IL28B **(282617), **IL1A **(3552), **CCL3 **(6348), **CCR7 **(1236), **CD274 **(29126), **STAT4 **(6775), **SOCS1 **(8651), **INDO **(3620), **ICAM1 **(3383), **CD83 **(9308), **MHC**(HLA-DRB5 (3127), HLA-DRB4 (3126), HLA-DRB3 (3125), HLA-DRB1 (3123), HLA-DRA (3122)).

TLR4	100	37	**Transduction modules: PI(4,5)P2, IRAK2 **(3656), **IRAK3 **(11213), **MAP3K7IP3 **(257397), **The protein family dynamin **(DNM3 (26052), DNM2 (1785), DNM1 (1759)), **RIPK3 **(11035), **TNKS **(8658), **AZI2 **(64343), **TBKBP1 **(9755). Outcomes: **IL1A **(3552), **IL10 **(3586), **IL12A **(3592), **IL12B **(3593), **IL15 **(3600), **IL23A **(51561), **LTA **(4049), **CCL19 **(6363), **TNFSF10 **(8743), **IRF1 **(3659), **IRF3 **(3661), **IRF5 **(3663), **IRF7 **(3665), **IRF9 **(10379), **CXCL10 **(3627), **MHC **(HLA-E (3133), HLA-DMB (3109), HLA-DOA (3111), HLA-DPA1 (3113), HLA-DPB1 (3115), HLA-DQA1 (3117), HLA-DQB1 (3119), HLA-DRA (3122), HLA-DRB1 (3123), HLA-DRB3 (3125), HLA-DRB4 (3126), HLA-DRB5 (3127)).

TLR5	52	25	**Transduction modules: The protein family PI3K **(PIK3R2 (5296), PIK3CG (5294), PIK3CD (5293), PIK3CB (5291), PIK3CA (5290), PIK3C3 (5289), PIK3C2G (5288), PIK3C2B (5287), PIK3C2A (5286), PIK3R1 (5295), PIK3R5 (23533), PIK3R6 (146850), PIK3R4 (30849), PIK3R1OS (404543), PIK3R3 (8503)), **PRKD1 **(5587), **AKT1 **(207). **Outcomes: CXCL2 **(2920), **IL18 **(3606), **CCL20 **(6364), **IL10 **(3586), **IL12A **(3592), **IL12B **(3593), **IL18 **(3606), **CCL2 **(6347).

TLR7	56	7	**Transduction modules: MEF2C **(4208), **PIK3CA **(5290), **PIK3CB **(5291), **PIK3CG **(5294), **IRF4 **(3662). **Outcomes: IL12A **(3592), **IL12B **(3593).

TLR8	52	5	**Transduction modules: MEF2C **(4208), **IRF1 **(3659). **Outcomes: IL12A **(3592), **IL12B **(3593), **NOS2 **(4843).

TLR9	54	21	**Sensing module: UNC93B1 **(81622). **Transduction modules: PIK3CD **(5293). **Outcomes: MHCII **(HLA-DMB (3109), HLA-DOA (3111), HLA-DPA1 (3113), HLA-DPB1 (3115), HLA-DQA1 (3117), HLA-DQB1 (3119), HLA-DRA (3122), HLA-DRB1 (3123), HLA-DRB3 (3125), HLA-DRB4 (3126), HLA-DRB5 (3127)), **CD80 **(941), **CD83 **(9308), **CD86**(942), **CD40**(958), **CCL3 **(6348), **CXCL10 **(3627), **ICAM1**(3383), **CCR7 **(1236).

To facilitate meaningful analysis of "omics" data, the pathways in DC-ATLAS are organized in a modular structure. Every signaling cascade downstream a specific receptor was divided into 3 types of modules in which the very last component of one module is also the first component of the subsequent module. The first type of module is the receptor and sensing module and comprises component(s) of the pathway directly interacting with the stimulus. The second transduction module, encompasses all components transducing the incoming signal from the sensing module downstream to the nucleus. This module generally starts with a molecule interacting with the receptor and ends with a transcription factor. The third and final module is the outcome module: it describes the end result of the signaling process. This last module begins with a transcription factor and includes target genes whose expression is altered after activation of the receptor. Complex cell functions, such as apoptosis, migration and differentiation are also described as outcomes.

**Figure 2:**
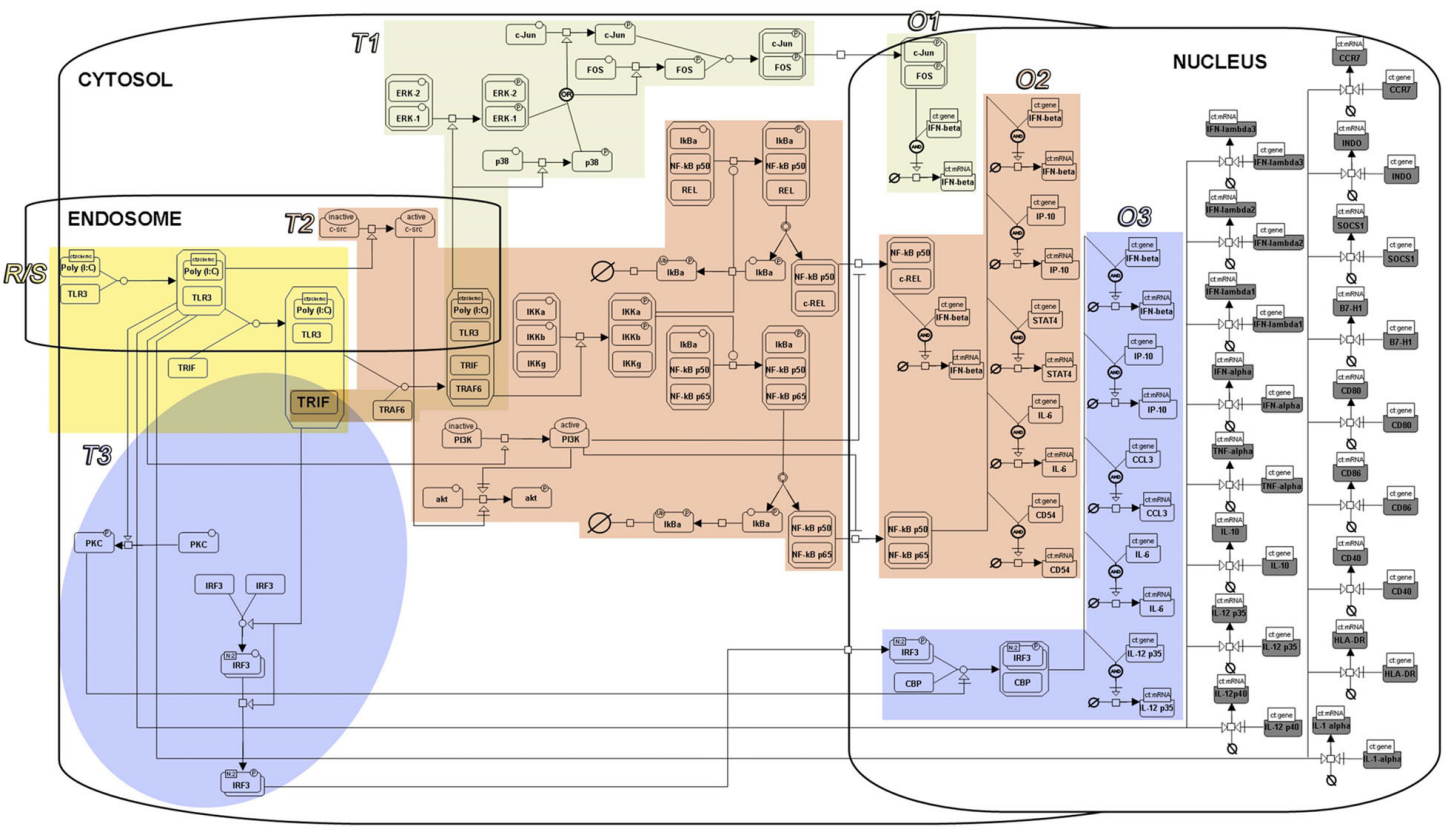
**SBGN representation of the DC-ATLAS human TLR3 signaling pathway**. The different modules are represented: The Receptor/Sensing module (R/S, in yellow), the different Transduction modules (T1, light grey; T2, pink; T3, light blue) and the Outcome modules (O1, O2, O3). The outcomes modules are colored in the same way of the transduction module by which they derived. TRIF is the key element shared by each transduction module. The outcome module in grey represents the genes which expression is proven in human DCs but the transcription factor that regulated their expression is not clearly identified.

According to the previous module definition, the pathways in DC-ATLAS may contain more than one of each type of modules. As an example, Figure 2 shows the modular structure of the TLR3 pathway curated for DC-ATLAS. In this pathway, one receptor/sensing module and three transduction modules leading to the activation of three critical transcription factors, IRF3, NF-kB and AP-1 have been identified.

The modules, as we defined them, have been subsequently tested using gene expression data as described in the following paragraphs. It should be emphasized that the transduction modules are not independent but are highly interconnected and partially overlapping. Furthermore, a given outcome may result from activation of more than one transduction module.

**Figure 3:**
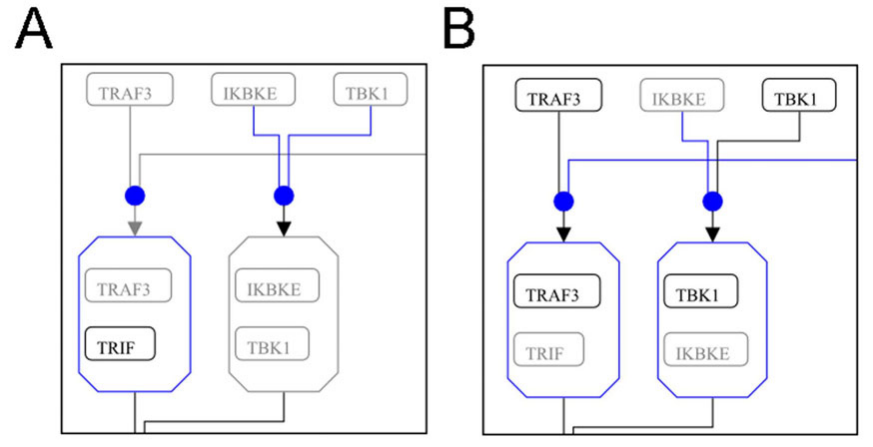
**Presence or absence of specific Toll-like receptor (TLR) 3 pathway elements in different cell types according to the currently available knowledge**. (A) Section of TLR3 pathway described in DCs; (B) Section described in macrophages. Grey elements are members of the pathway whose presence has not been demonstrated in the specific cell type (DCs and macrophages, respectively). Blue elements and lines indicate reactions and entities that depend on absent (grey) members and thus may not occur. The complete pathway representations are available as Additional file 3 Figure Sl and Additional file 4 Figure S2.

The data format we used to describe the pathway allowed us to depict interactions in the cellular organelles where they occur as well as to specifically mark genes and interactions according to the biological system (e.g., cell type and species) where they took place. Thus, we were able to create a map of the TLR3 pathway for example clearly showing which genes and interactions were described in DCs and which were not (Figure 3, Additional file 3 Figure S1 and Additional file 4 Figure S2).

Overall, these results provide strong support for the importance of curating a pathway with the final aim of defining all interactions and nodes occurring in a specific species, cell type and compartment.

### DC-ATLAS is a powerful tool to dissect TLR specific contributions and to analyze time course related responses

To address the importance of the modular structure of the DC-ATLAS and its statistical approach in dissecting the contribution of TLRs, we performed a time-course transcriptional analysis of moDCs stimulated with LPS and risiquimod (R848) that respectively activate TLR4 and TLR7/8. We calculated pathway signatures for each of these datasets and subsequently clustered resulting pathways (see Methods).

**Figure 4:**
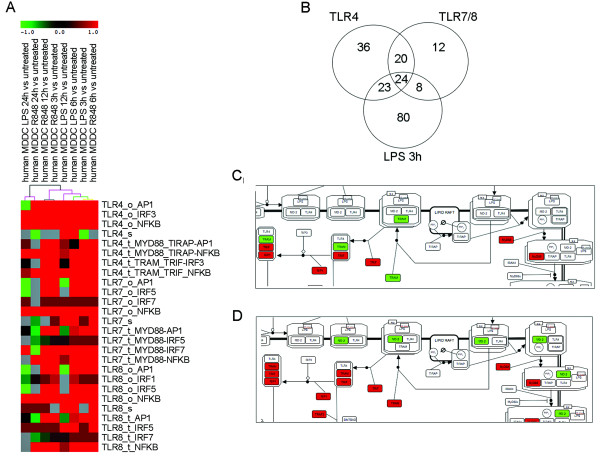
**Pathway analysis on microarray data on DCs stimulated with R848 and LPS using DC-ATLAS pathways**. (A) Section of clustering of PEF and score using Euclidean distance using support trees on DCs stimulated with R848 and LPS for different periods of time: 3, 6, 12 and 24 hours. Colored spots indicate significant up- (red) or down- (green) regulation. The colors of the dendrogram indicate the percentages of the tree support (significance), from 50% (pink) to 100% (black). The pathways are named as name of receptor_module_adaptor-TF involved. "s", "t" and "o" indicated sensing, transduction and outcome module, respectively. The total matrix used for clustering is available as Additional file 5. (B) Interpolation of DEGs of DCs in response to 3 hours LPS stimulation, the specific agonist of TLR4 signaling, with the gene lists representative of elements participating in TLR7/TLR8 pathways and representative of elements composing TLR4 pathway (in the Venn diagram indicated as TLR7, TLR8 and TLR4, respectively). (C) SBGN representation of the TLR4 pathway highlighting gene regulation upon 3 hours-stimulation with LPS. Red indicates up-regulation while green signifies down-regulation. (D) SBGN representation of the TLR4 pathway highlighting gene regulation at 6 hours. The full figures are available as Additional file 6 Figure S3 and Additional file 7 Figure S4, respectively.

By clustering pathway signatures using publically available TLR pathways, it proved virtually impossible to obtain information of individual potentially affected elements within the TLR pathway, despite clear up-regulation at the pathway level. Instead, clustering of DC-ATLAS based results readily showed a separation of different stimulatory conditions (Figure 4A). The total matrix used for clustering is available as Additional file 5.

As we expected both the TLR7/8 and TLR4 modules were affected upon specific stimulation, with R848 and LPS respectively [24]. At early time points, analysis allowed appreciation of activation of specific signal transduction modules while at later time points, outcome modules were clearly activated and sensing modules were down-regulated or not affected, indicating a general feedback regulation in fully matured DCs. At this stage, DCs have committed to their fate and decided how to respond to a specific stimulus making some of its sensing receptors redundant. Despite the overlap between signaling from both receptors, the cluster analysis indicated how DCs stimulated for 6 hours with R848 behave similarly to cells stimulated for 3 hours with LPS, underlining a slower activation of the signaling through TLR7/8, perhaps due to their intracellular localization in the endosome. At 24 hours, when the DC maturation process is completed, the profiles of the pathway signatures are more similar between the two stimuli.

Also in time course experiments, the modular structure of DC-ATLAS allows to appreciate time-dependent changes in expression providing a more informative analysis. The TLR4-sensing module is repressed at 3 hours of LPS stimulation. After 12 hours of stimulation, the MyD88 dependent signaling module is less abundant when compared to MyD88 independent transduction modules during the earliest time points. As can be seen in Figure 4A, after 24 hours of LPS stimulation, the outcome modules activated by AP-1 become repressed. Similarly, upon R848 stimulation, the sensing module is over-represented at early time points and switched-off later on. After 24 hours, several parts of the signal transduction module are repressed as well as the outcome module indicating a commitment of the cells or a feedback regulation. Together, these observations nicely demonstrate that, using DC-ATLAS, we can follow the signal, as a temporal series of discrete events across all the modules, from sensing to outcome trough the transduction part.

As can be seen from the analysis, in addition to a single TLR specific pathway, a number of other TLR pathways can be affected by the stimuli used. This is because several of the TLR pathways, such as the TLR4 and TLR7/8 pathways, share some elements, although this does not necessarily mean that their engagement leads to identical outcome. When analyzing the LPS dataset at 3 hours, 135 genes were found to be differentially expressed within the DC-ATLAS pathways and 47 of them belonged to the TLR4 signaling pathway (Figure 4B). Among these, 24 were shared with TLR7/8 pathways, while 23 elements were assigned specific for TLR4 (Figure 4B).

Given the modular structure and DC specific annotations of DC-ATLAS we can also evaluate individual elements involved in TLR specific signaling. For example, we mapped differentially expressed genes upon 3 hours-LPS stimulation from our data set to the TLR4 pathway (Figure 4C and Additional file 6 Figure S3). Using this map and the output of the pathway analysis, it becomes now possible to appreciate the entire flow of the signal starting from the receptor till the final activation of the transcription of specific genes inside the nucleus. It is well established that TLR4 engagement can result in different signaling, dependent on the adaptors recruited [25,26]. The signal either starts from MyD88 and the MyD88-like adapter (TIRAP), or from the TIR-domain-containing adapter-inducing interferon-beta (TRIF, also shared by TLR3) and the TRIF-related adapter molecule (TRAM). We observed that this is highly time dependent as the signal trough TRAM at 3 hours was still down-regulated (Figure 4C) and became up-regulated at 6 hours after stimulation (Figure 4D and Additional file 7 Figure S4).

These results thus illustrate that the modular structure of DC-ATLAS allows a better and more detailed understanding of TLR mediated signaling in time course experiments.

### DC-ATLAS can discriminate between species-specific pathways

**Figure 5:**
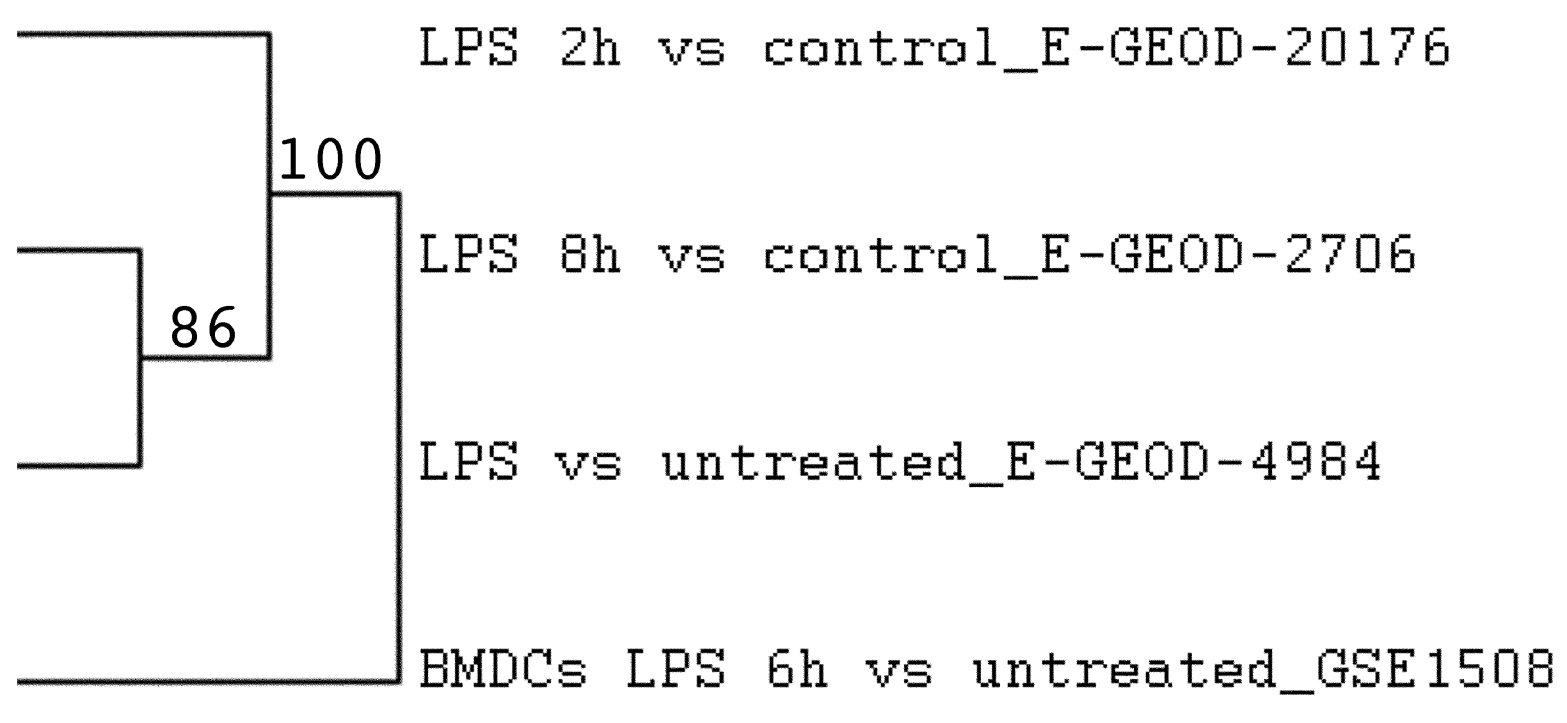
**Pathway analysis on microarray data on human or mouse DCs stimulated with TLR ligands**. Dendogram of PEF cluster and score using Euclidean distance using support trees on human moDC or mouse bone marrow derived DCs (BMDCs) stimulated with LPS. The numbers next to the tree indicate the support (significance): higher values mean higher significance. The total matrix used for clustering is available as Additional file 8.

Currently, studies on mouse DCs outnumber those on human cells; however, comparisons between mouse and human models have been somewhat biased due to biological differences between both species [27] as well as differences in the origin of the material used to study DCs, e.g. bone marrow derived mouse DCs (BMDC) versus monocyte derived human DCs (moDCs). The species-specific curation of the DC-ATLAS pathways allowed us to highlight the differences between mouse and human model DC signaling in response to similar stimuli (Figure 5). When we perform a pathway and cluster analysis on publically available human moDC- (GSE2706, GSE4984) and mouse BMDC- (GSE15087) datasets, we could clearly identify a different profile from the mouse data when compared with the human data, even though they were both stimulated with LPS (Figure 5), although we should take into account they were derived from different progenitors. The total matrix used for clustering is available as Additional file 8.

## Discussion

An immune response forms a complex biological system with many possible inputs, influences and outcomes, in which DCs play a critical role. The relationship among the different immune cells subsets and model systems are currently under active debate, highlighting the importance of annotating signaling pathways in respect of both the cell type and the species in which the pathway was found.

Zanoni et al. reviewed the divergent responses of DCs and macrophages upon LPS [28], and despite the evolutionary conserved mechanisms between the human and murine immune system, an increasing number of studies demonstrate that several cell-specific differences in signal transduction exist [29].

Studying DCs using an immune systems biology approach facilitated by DC-ATLAS, holds promise to dissect the integrated signals from these cells. This allows us to build models of the complex process of DC regulation and generate predictions and hypotheses about DC function under physiological and pathological conditions. However, the road forward is not without obstacles. There is a strong need of a greater coverage of network data, improved accuracy and standardization of annotation. The extraction of signal transduction maps from gene expression data requires well-structured pathway definitions. Similarly to the recently published DC pathway map [9], DC ATLAS is an integrated project incorporating both immunology and bioinformatics, focused on signaling pathways in DCs. Yet, with respect to other existing resources, DC-ATLAS denotes major advancements. It describes the reactions based on consensus reached by a large number of leading European immunological scientists with expertise in DCs. The pathways represent a valuable tool to emphasize established facts as well as to highlight limitations in our knowledge with respect to the hierarchy of events leading to effective immune responses. DC-ATLAS is the first SBGN compliant pathway databases implemented using the novel Biological Connection Markup Language (BCML). DC-ATLAS integrates a detailed pathway information with experimental data allowing data analysis in a DC-specific manner. It is the first example of a modular approach to describe signal transduction pathways. Here, sets of reactions that participate in a common regulatory unit were functionally categorized as part of what we defined as a "module". The interconnected modules, which describe the DC-ATLAS pathways from receptors to effectors via signal mediators, overcome a major limitation of the current pathway structures, in which specific events are masked by a plethora of generic interactions.

The presented results show how DC-ATLAS allows temporal dissection of events within the signal, represented by 3 different modules grouping the sensing/receptor, the transduction of the signal and outcome pool. The pathway analysis perturbations, with these modular pathways, visualize the signal propagation, keeping track of the flow of information.

As demonstrated by LPS and R848 stimulation of DCs (Figure 4A), the upregulation of outcome modules often corresponds to down-regulation of sensing/receptor modules at later time-points. This reverse regulation of modules has to be interpreted as the presence of a negative regulatory feedback loop from the outcome to the sensing module. This retrograde regulation is well documented in literature [30]. Sensor proteins undergo a rapid turnover and the regulation of their abundance is required to maintain the plasticity of the system. As a consequence, in TLR mediated signaling the transcription of genes encoding receptor proteins appears activated in the first 1-3 hours following stimulation, and down-regulated as soon as the cells becomes committed. Interestingly, key elements of the signal transduction module remain transcriptionally controlled despite the fact that propagation of these signals depend on events such as phosphorylation or protein binding, in agreement with a recent report by Buschow et al. [31].

This type of dissection of the flow of information described as changes in gene expression is made possible solely by the use of BCML. It provides a suitable format to store the information collected and organized by the curators and to build the modular pathways. Our goal was to provide a data format that was easily extensible and manipulable for computational analyses, but at the same time intuitive and user friendly for both cell biologist and immunologist communities. BCML satisfies these needs thanks to its flexibility, which permits its use in computational analysis and in the conversion to a SBGN compliant graphical map. The possibility of filtering permits the creation of "customized" networks, better suited to identify specific biological problems or to highlight gaps in current knowledge. At this moment, BCML only covers the SBGN Process Description. In the near future we will also integrate. the SBGN Entity Relationship and Activity Flow representation. in order to provide a complete representation of SBGN at the data level.

We developed our own format since neither of the many existing formats such as KGML, BioPAX [32] or SBML [33] were suitable: some formats lacked a biological graphical representation (SBML), while others were not SBGN compliant (BioPAX, KGML).

Being SBGN compliant and machine readable, BCML provides a convenient and precise way to represent biological pathways, in an intuitive and user friendly format to both the biologist and the bioinformatician.

Because of the more refined modular description of pathways in DC-ATLAS, the results of statistical analysis are much improved with respect to the results one can obtain from existing pathway databases.

The modular pathway of DC-ATLAS allows more accurate capture of signaling pathways. In many cases pathway modules are not specific for just one stimulus. This overlap is expected given the combinatorial nature of the module structure and definition that describes the biological nature of signal transduction in DCs. This is an important feature, as the decision making process of DCs often integrates signaling from multiple receptors and temporal integration of multiple sets of stimuli.

## Conclusions

New computational methods such as DC-ATLAS will contribute to fill in current gaps in the analysis of genomic data. Furthermore, the ability of DC-ATLAS to identify gaps in our current knowledge will foster future research within the immune system, and lead to the design of novel experiments aimed at reconciling interactions and findings documented in human and mouse DCs. In addition, DC-ATLAS will establish the relationships between pathways operating within DCs and other cell types of different species.

In conclusion, DC-ATLAS provides a knowledge base on DC biology with the potential to decipher the complex network of interactions occurring within these cells in response to activation stimuli. This knowledge allows the conversion of genomic research into accurate and robust biological hypotheses. Extracting results from large expression datasets using DC-ATLAS will enable us to validate experimentally defined pathways and generate signatures that will serve as valuable tools in the design of new strategies for DC-based immunotherapy.

## Methods

### Pathway curation process

We selected several pathways of interest for immunology, in particular for DC activation, as TLRs pathways and curated them. We handled the currently available information from the literature and from public available pathway databases, such as KEGG, Reactome and GenMAPP, evaluating the quality of the data, as well as experimental evidence generated in our laboratories to curate/design the pathways in a cell specific manner. Detailed information are available on line as Additional file 1.

### Pathway representation

Pathways were drawn following the SBGN Process Description (PD) 1.1 specification [10]. Following curation, pathway were represented using the Biological Connection Markup Language (BCML), a machine-readable data format built on the SBGN specification, including all the information collected by the curators (Additional Material: Curation Process). The BCML representation was then transformed to a graphical map. Detailed information are available on line as Additional file 2.

The DC-ATLAS pathways were also represented in GPML (using an in-house modified version of the PathVisio program [34]) and INOH format (http://www.inoh.org), (http://www.dc-atlas.net).

### DCs transcriptional analysis to pure TLR ligands

Peripheral blood mononucleated cells (PBMC) were isolated from buffy coat blood sample from healthy donors from the Transfusion Unit Erlangen hospital (Erlangen, Germany) by Ficoll-Hypaque density gradient centrifugation (Biochrom AG). The experimental plan was approved by the local Ethical Committee, and informed consent was obtained from all donors. Monocytes were isolated from low density PBMCs by magnetic enrichment with anti-CD14 beads (Miltenyi Biotec). Cells were cultured in the presence of granulocyte macrophage colony stimulating factor (GM-CSF, 800 U/ml) and recombinant IL-4 (1000 U/ml) for 6 days to allow DC differentiation [35]. 2 × 10^6 ^DCs were cultivated with LPS (100 ng/ml) or R848 (2,5 μg/ml) or without any stimuli. After 3, 6, 12 and 24 hr, cells were collected. RNA preparation, labeling with Cy5, hybridization on a Human HT12 array (Illumina), and scanning were performed according to the Illumina reference protocols.

### Array pre-processing

Bead-summary data saved from Illumina BeadStudio was pre-processed in several steps. Firstly, the background signal was assessed and corrected using the intensity signal from the control probes present on the array, then quantile normalization was performed. In addition to background correction, Illumina probe identifiers were converted to nucleotide universal IDentifiers (nuIDs) [36] specific for the nucleotide sequence of each probe. The computation was performed using the lumi package [37], written in the R programming language.

Microarray data have been submitted to the Array Express repository, with the accession number E-MTAB-448.

### Public data sets and data preprocessing

Publicly available data sets were retrieved from the Gene Expression Omnibus (GEO) database. After retrieving, they were normalized with the Robust Multi-array Average (RMA) method [38] in the case of Affymetrix data, and with quantile normalization for other array platforms. Affymetrix data were also re-annotated with the most recent data available following the procedure by Dai et al. [39]. Preprocessing was performed with the RMAExpress software (Affymetrix data; http://rmaexpress.bmbolstad.com) or with the R programming language (other platforms).

### Pathway analysis

Pathway analysis was performed with PathStudio (Beltrame *et al.*, unpublished data), over the compendium of DC-ATLAS TLR pathways. Prior to the analysis, microarray raw data were transformed into absolute-scale values and processed following the procedure outlined by [40]: firstly, ratios between each treated condition and the unstimulated controls were calculated. Then, in an effort to reduce inter donor variability, the mean of the ratios for all replicates in a specific condition was calculated. The resulting ratios were used to perform pathway analysis using the Fisher's Exact Test and the resulting signed p-values were transformed into Pathway Enrichment Factors (PEFs), applying a scoring metric rather than the Fisher's Exact Test transformed p-value for pathways with less than five elements. PEFs were clustered using multiscale bootstrap resampling [41] over 1000 iterations.

## Abbreviations

BCML: Biological Connection Markup Language; BMDC: bone marrow derived dendritic cell; CLR: C-type lectin like receptor; DC: dendritic cells; GEO: Gene Expression Omnibus database; LPS: lipopolysaccharides; moDC: monocyte derived dendritic cells; NLR: NOD-like receptor; PD: Process Description; PEF: pathway enrichment factor; PRR: pathogen recognition receptor; RLR: RIG-I-like receptors; R848: risiquimod; RMA: Robust Multi-array Average; SBGN: Systems Biology Graphical Notation; TLR: Toll like receptor.

## Competing interests

The authors declare that they have no competing interests.

## Authors' contributions

DC wrote the manuscript, coordinated and supervised the project and jointly conceived the study with JMA and CGF. SIB, SG, MCG, WR, AB, CDF, UDO, IDS, EG, FG, MK, MKu, AL, CJMKM, NM, RO, PP, ER, GS, VS, IS, MGT, IZ, RZ, curated and edited both pathways and vocabularies and contributed to the project's creation and development. DR, LB, LR and EC edited the pathways and vocabularies, developed the bioinformatic infrastructure and contributed to the manuscript revision. SS performed time-course experiments. RB, MB, AS, MG, RP and SD contributed to the development of the bioinformatic infrastructure. All authors read and approved the final manuscript.

## Supplementary Material

Additional file 1 **Curation process description**. The procedure of curation taken towards the reconstruction and editing of the public available DC pathways and the *de novo *curation of pathways not previously presented in public databases. Click here for file

Additional file 2 **BCML Description**. The definition of the Biological Connection Marked Language and the description of its features. Click here for file

Additional file 3 **Figure S1: SBGN representation of the TLR3 signaling pathway highlighting the reactions that occur only in dendritic cells**. Black elements are entities whose presence has been demonstrated in dendritic cells (DCs); grey elements indicate entities whose presence has not been demonstrated in DCs. Blue elements highlight reactions that depend on non present (grey) elements and thus may not occur. Click here for file

Additional file 4 **Figure S2: SBGN representation of the TLR3 signaling pathway highlighting the reactions that occur only in macrophages**. Black elements are entities whose presence has been demonstrated in dendritic cells (DCs); grey elements indicate entities whose presence has not been demonstrated in DCs. Blue elements highlight reactions that depend on non present (grey) elements and thus may not occur. Click here for file

Additional file 5 **Pathways analysis results of LPS vs R848 comparison: input matrix**. A matrix of Pathway Enrichment Factors (PEFs) obtained from the transformation of the signed p-values derived from the pathway analysis. This matrix can be used for clustering using multiscale bootstrap resampling or other methods. Click here for file

Additional file 6 **Figure S3: Enriched genes found to be part of TLR4 signaling upon LPS stimulation superimposed to the SBGN pathway map**. Differentially expressed genes of DCs stimulated for 3 hours with LPS present in the TLR4 signaling superimposed to the pathway map. Red nodes indicate that the respective genes are up-regulated, and green nodes indicate down-regulated genes. Click here for file

Additional file 7 **Figure S4: Enriched genes found to be part of TLR4 signaling upon LPS stimulation superimposed to the SBGN pathway map**. Differentially expressed genes of DCs stimulated for 6 hours with LPS present in the TLR4 signaling superimposed to the pathway map. Red nodes indicate that the respective genes are up-regulated, and green nodes indicate down-regulated genes. Click here for file

Addtional file 8 **Pathways analysis results of human vs mouse comparison: input matrix**. A matrix of Pathway Enrichment Factors (PEFs) obtained from the transformation of the signed p-values derived from the pathway analysis. This matrix can be used for clustering using multiscale bootstrap resampling or other methods. Click here for file
