# Supplementary material for: DC-ATLAS: a systems biology resource to dissect receptor specific signal transduction in dendritic cells
Source: Immunome Res. 2010 Nov 19;6:10. doi: 10.1186/1745-7580-6-10 (PMC3000836; doi:10.1186/1745-7580-6-10)
Supplement: Additional file 1 — Curation process description. The procedure of curation taken towards the reconstruction and editing of the public available DC pathways and the de novo curation of pathways not previously presented in public databases. [file 1745-7580-6-10-S1.PDF]

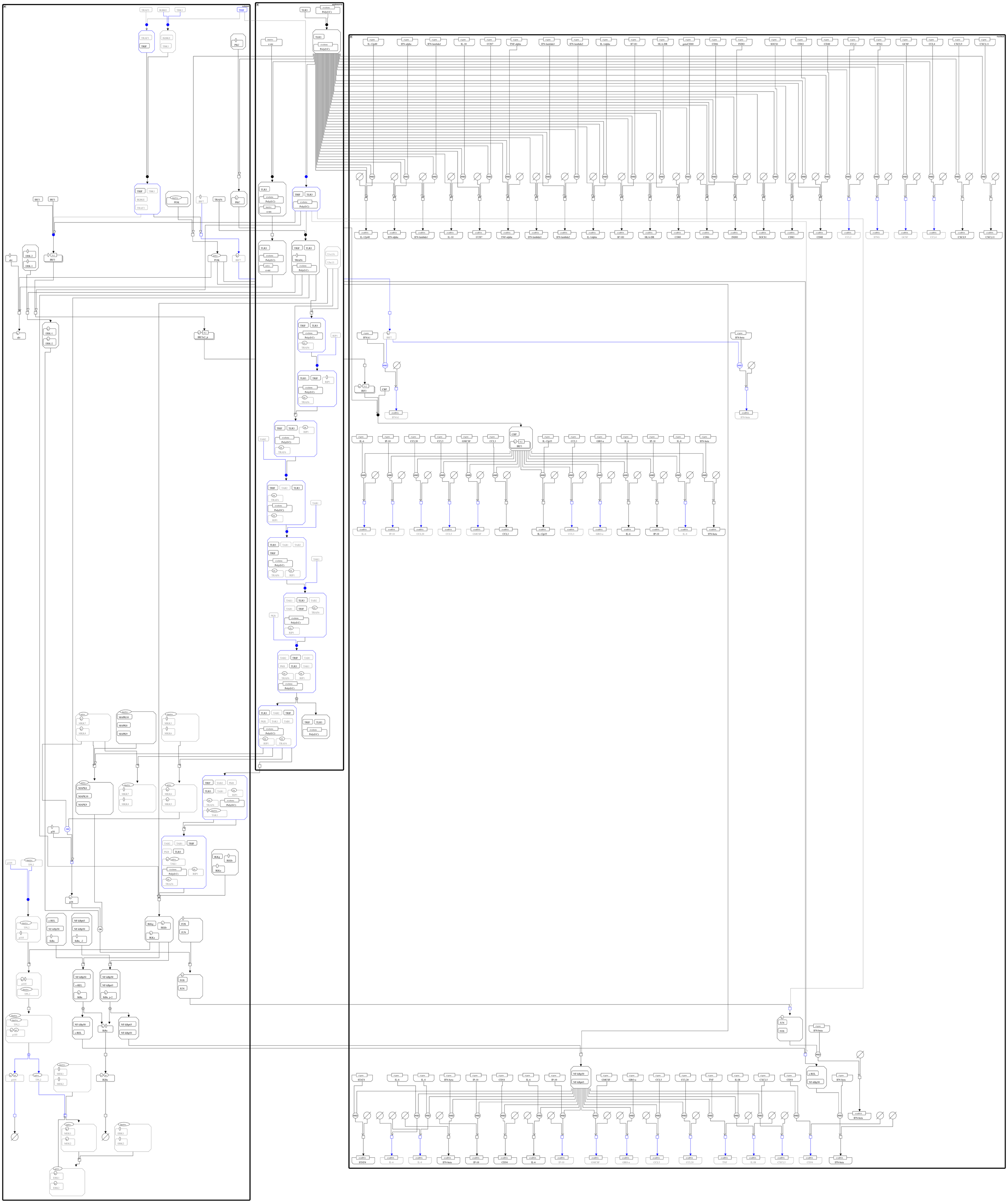

TLR3
TRIF
Poly(I:C)
TRAF3
IRF3
PI3K
PKC
IRF7
TRAF6
RIP1
IKKα
IKKβ
IKKγ
NF-kBp50
NF-kBp65
c-REL
IkBα
p38
FOS
JUN
IFN-beta
IL-6
IP-10
CCL5
CCL20
GMCSF

Supplementary Figure 1. SBGN representation of the TLR3 signaling pathway highlighting the reactions that occur only in dendritic cells. Black elements are entities whose presence has been demonstrated in DCs; grey elements indicate entities whose presencne has not been demonstrated in DCs. Blue element indicate reactions that depend on non present (grey) elements and thus may not occur.
