# Supplementary material for: DC-ATLAS: a systems biology resource to dissect receptor specific signal transduction in dendritic cells
Source: Immunome Res. 2010 Nov 19;6:10. doi: 10.1186/1745-7580-6-10 (PMC3000836; doi:10.1186/1745-7580-6-10)
Supplement: Additional file 2 — BCML Description. The definition of the Biological Connection Marked Language and the description of its features. [file 1745-7580-6-10-S2.PDF]

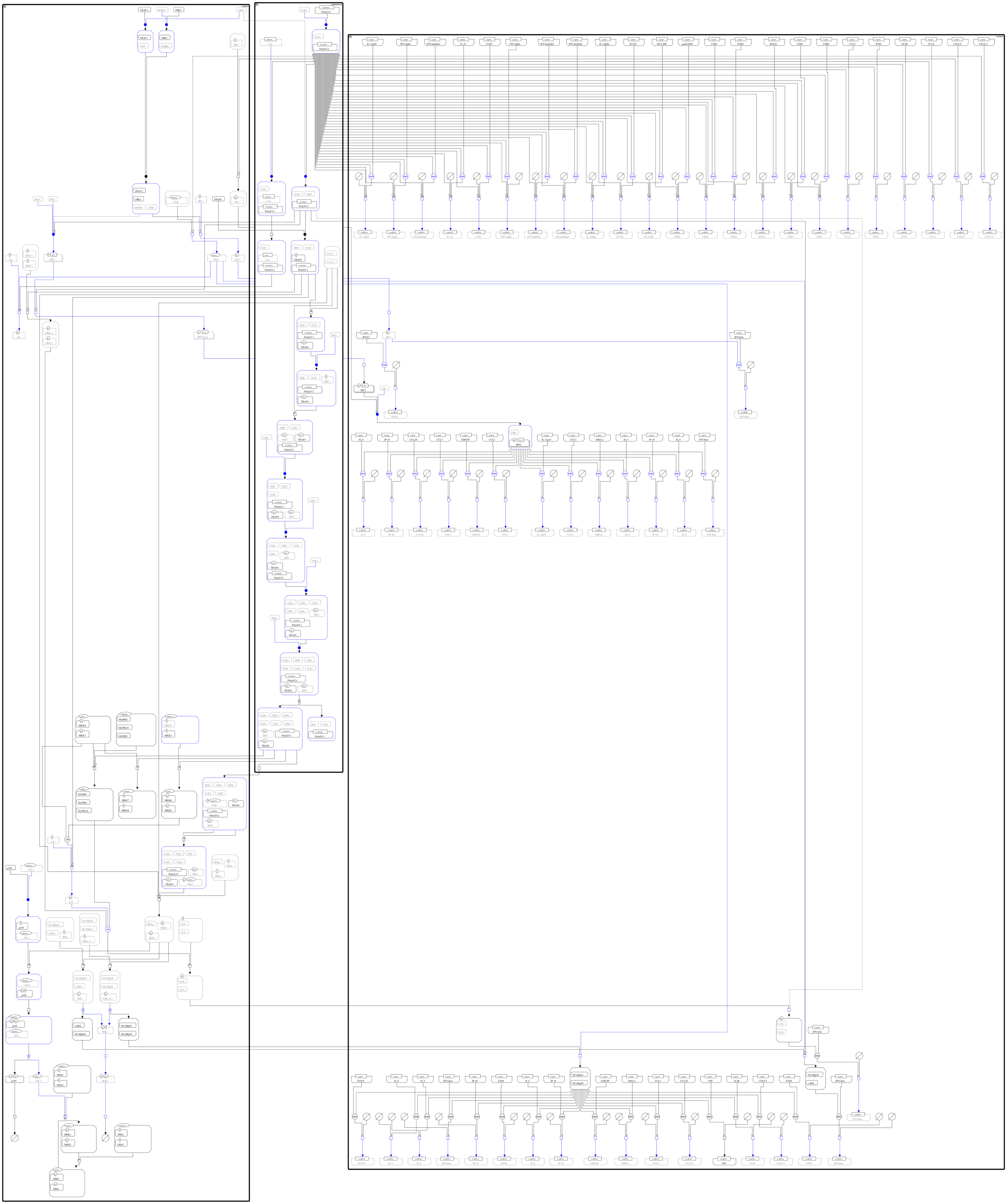
cytosol
endosome
nucleus
Poly(I:C)
IL-12p40
IFN-alpha
IFN-lambda1
IL-10
CCR7
TNF-alpha
IFN-lambda3
IFN-lambda2
IL-1alpha
B7-H1
HLA-DR
geneCD80
CD86
INDO
SOCS1
CD83
CD40
CCL2
IFNG
GCSF
CCL4
CXCL9
CXCL11

Supplementary Figure 2. SBGN representation of the TLR3 signaling pathway highlighting the reactions that occur only in macrophages. Black elements are entities whose presence has been demonstrated in macrophages; grey elements indicate entities whose presencne has not been demonstrated in macrophages. Blue element indicate reactions that depend on non present (grey) elements and thus may not occur.
