# Supplementary material for: DC-ATLAS: a systems biology resource to dissect receptor specific signal transduction in dendritic cells
Source: Immunome Res. 2010 Nov 19;6:10. doi: 10.1186/1745-7580-6-10 (PMC3000836; doi:10.1186/1745-7580-6-10)
Supplement: Additional file 3 — Figure S1: SBGN representation of the TLR3 signaling pathway highlighting the reactions that occur only in dendritic cells. Black elements are entities whose presence has been demonstrated in dendritic cells (DCs); grey elements indicate entities whose presence has not been demonstrated in DCs. Blue elements highlight reactions that depend on non present (grey) elements and thus may not occur. [file 1745-7580-6-10-S3.PDF]

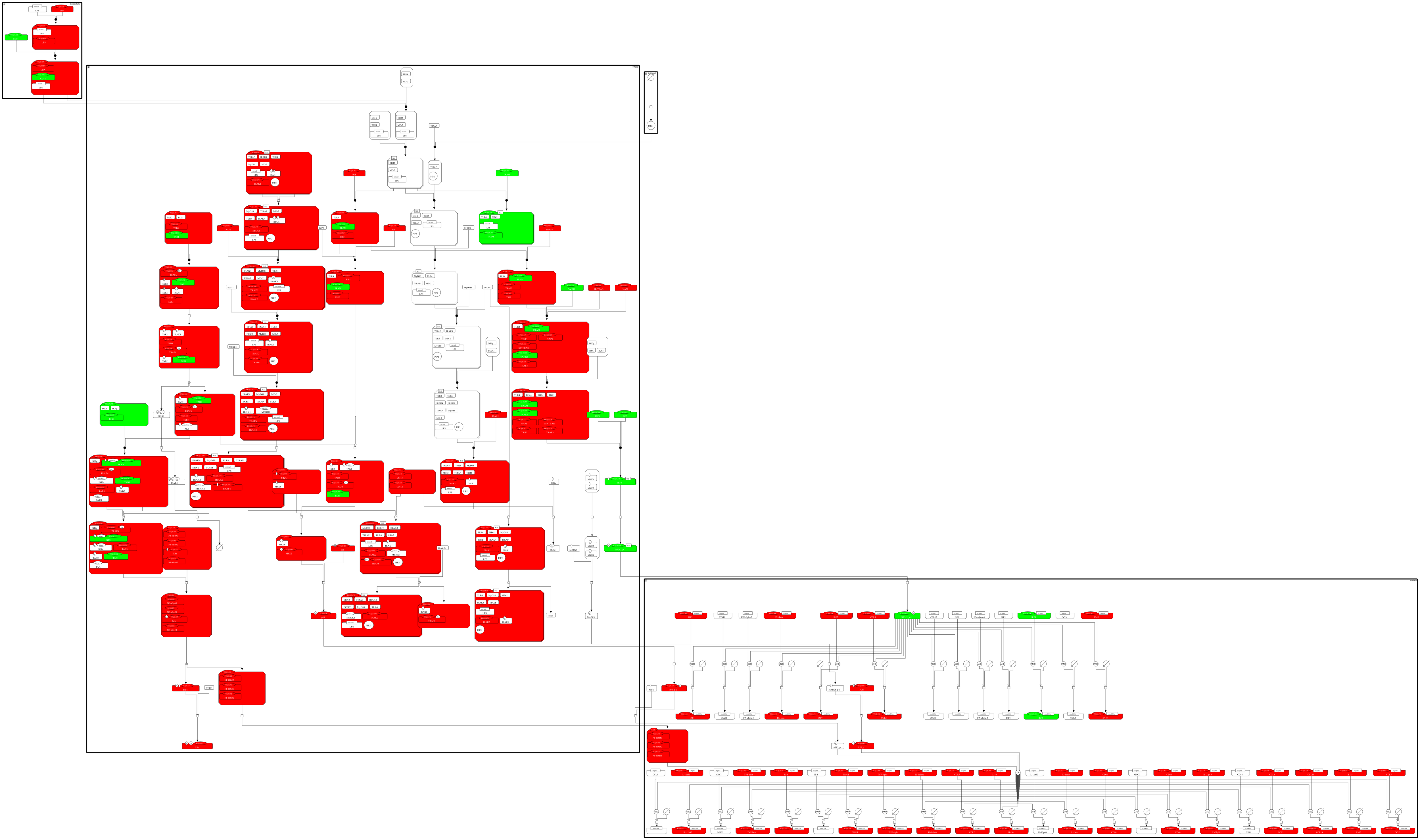

Supplementary Figure 3. Enriched genes found to be part of TLR4 signaling upon LPS stimulation superimposed to the SBGN pathway map. Differentially expressed genes of DCs stimulated for 3h with LPS present in the TLR4 signaling superimposed to the pathway map. Red nodes indicate that the respective genes are up-regulated, and green nodes show down-regulated genes.
