# Supplementary material for: DC-ATLAS: a systems biology resource to dissect receptor specific signal transduction in dendritic cells
Source: Immunome Res. 2010 Nov 19;6:10. doi: 10.1186/1745-7580-6-10 (PMC3000836; doi:10.1186/1745-7580-6-10)
Supplement: Additional file 5 — Pathways analysis results of LPS vs R848 comparison: input matrix. A matrix of Pathway Enrichment Factors (PEFs) obtained from the transformation of the signed p-values derived from the pathway analysis. This matrix can be used for clustering using multiscale bootstrap resampling or other methods. [file 1745-7580-6-10-S5.DOC]

**Curation Process**

Critical signalling pathways in dendritic cells have been selected and curated using information present in databases, literature, as well as experimental evidence gathered in the laboratories of the researchers involved in DC-ATLAS project.

The curators described each pathway by creating a text file, a gene list, and a graphical representation available at http://www.dc-atlas.net. The text file described the pathways conforming to the BCML data format and the controlled vocabularies.

We built hierarchical structured controlled vocabularies by both: i) changing previously existing terms from public databases, as well as ii) adding missing terms that our community felt were needed.

Six different vocabularies, containing at least 400 ontological terms, mainly from Open Biological Ontologies (OBO) (1) have been assembled as follows:

• Reactant: terms used to name the pathway nodes. The terms refer to the generic name of protein or chemical involved in a pathway.

• Reaction: terms used to identify the type of interaction involved in the pathway (phosphorylation, dephosphorylation, ubiquitination, etc).

• Cell type: the type of the cell in which the pathways are described (Monocyte derived DCs, plasmacytoid DCs, etc) .

• Organism: terms used to identify the species in which the pathways are described (*Homo sapiens, Mus musculus*, etc).

• Location: terms used to specify the cellular part where the pathway/reaction happen (cytoplasm, nucleus, membrane, endosome, etc).

• Experimental design: the type of experiment undertaken in order to demonstrate the interactions between genes in the pathway.

Genes in the gene lists describing each pathway will be annotated with their official HGNC (HUGO Gene Nomenclature Committee, www.genenames.org) (2) symbol and their Entrez Gene ID (www.ncbi.nlm.nih.gov/Entrez) (3).

Pathways were drawn following the SBGN Process Description (PD) 1.1 specification (4). Following curation, pathway were represented using the Biological Connection Markup Language (BCML), a machine-readable data format built on the SBGN specification. The BCML representation was then transformed to a graphical map.

The DC-ATLAS pathways were also represented in GPML (using an in-house modified version of the PathVisio program) (5) and INOH format (www.inoh.org) (http://www.dc-atlas.net). While the former allows simplicity and immediacy, the latter allows to appreciate the complexity and temporal events of the biological process.

The written and graphical pathway description provided the related bibliography used for the description of the pathways and annotated the species and the cell type where the information has been obtained, as well as the reaction subcellular localization.

**References**

**1.** Smith B, Ashburner M, Rosse C, Bard J, Bug W, Ceusters W, Goldberg LJ, Eilbeck K, Ireland A, Mungall CJ; OBI Consortium, Leontis N, Rocca-Serra P, Ruttenberg A, Sansone SA, Scheuermann RH, Shah N, Whetzel PL, Lewis S. **The OBO Foundry: coordinated evolution of ontologies to support biomedical data integration.** *Nat Biotechnol*. 2007, **25(11):**1251-5.

**2.** Bruford EA, Lush MJ, Wright MW, Sneddon TP, Povey S, Birney E. **The HGNC Database in 2008: a resource for the human genome.** *Nucleic Acids Res.* 2008, **36**(Database issue)**:**D445-8.

**3.** Maglott D, Ostell J, Pruitt KD, Tatusova T. **Entrez Gene: gene-centered information at NCBI.** *Nucleic Acids Res.* 2005, **33**(Database issue)**:**D54-8.

**4.** Le Novere N, Hucka M, Mi H, Moodie S, Schreiber F, Sorokin A, Demir E, Wegner K, Aladjem MI, Wimalaratne SM, Bergman FT, Gauges R, Ghazal P, Kawaji H, Li L, Matsuoka Y, Villeger A, Boyd SE, Calzone L, Courtot M, Dogrusoz U, Freeman TC, Funahashi A, Ghosh S, Jouraku A, Kim S, Kolpakov F, Luna A, Sahle S, Schmidt E, Watterson S, Wu G, Goryanin I, Kell DB, Sander C, Sauro H, Snoep JL, Kohn K, Kitano H: **The Systems Biology Graphical Notation.** *Nature biotechnology* 2009, **27:**735-741.

**5.** van Iersel MP, Kelder T, Pico AR, Hanspers K, Coort S, Conklin BR, Evelo C: **Presenting and exploring biological pathways with PathVisio.** *BMC bioinformatics* 2008, **9:**399.
