# Supplementary material for: DC-ATLAS: a systems biology resource to dissect receptor specific signal transduction in dendritic cells
Source: Immunome Res. 2010 Nov 19;6:10. doi: 10.1186/1745-7580-6-10 (PMC3000836; doi:10.1186/1745-7580-6-10)
Supplement: Additional file 6 — Figure S3: Enriched genes found to be part of TLR4 signaling upon LPS stimulation superimposed to the SBGN pathway map. Differentially expressed genes of DCs stimulated for 3 hours with LPS present in the TLR4 signaling superimposed to the pathway map. Red nodes indicate that the respective genes are up-regulated, and green nodes indicate down-regulated genes. [file 1745-7580-6-10-S6.DOC]

**Description of the Biological Connection Markup Language (BCML)**

BCML was defined using an XML Schema, supporting the complete SBGN PD definition, including entities, interactions, rules and restraints. The various SBGN elements are defined as XML tags, with additional properties stored in tag attributes.

In addition to a 1:1 implementation of the specification, BCML provides a series of optional features (defined as extensions of the main schema). First of all, BCML can include additional information on the entities that compose the network: each entity be described by a series of database identifiers, e.g., Entrez Gene or Uniprot accession numbers, and each species can have its independent set of identifiers. Furthermore, each entity or reaction can have a set of facts or “Findings” associated. “Findings” are collections of biological information that are relevant to that entity or reaction.

BCML also supports the functional module definition of DC-ATLAS through the use of the “MacroModule” tag.

Lastly, even though it is mostly a data container, BCML contains support for a number of graphical hints, such as border, background and text colors of the elements (while the original SBGN specification is monochromatic). These hints are recognized and processed by the tools that can read and parse BCML files.

All the additions to the SBGN specification are completely optional. It is important to note that the layerization of the format allows to add information without affecting the SBGN compliance.

**BCML software suite**

To support the use of the format, we developed a series of tools to ensure the proper description, manipulation and visualization of pathways using BCML mainly in five ways: validation, graphical representation, discriminative selection, incorporation of experimental measurements, and data analysis.

The *validator* ensures that the BCML file is well formed according to the XML specification, and secondly the network is examined for consistency, using rules and constraints defined by SBGN. The tool then reports to the user which elements are breaking the specification. Lastly, this tool ensures that identifiers for each element are unique and that isolated entities are not present.

The current implementation in the software suite supports the transformation of BCML files through a *converter* into GraphML, a widely used format for graph representation. BCML files converted to GraphML can be opened by programs such as the yEd graph editor, where they can be exported to vector graphs or bitmap images.

The *filter* permits the manipulation of the pathway data, creating a new network containing only elements with user-defined characteristics. When the discriminative selection is applied to the pathway, elements are marked as “included”, “excluded”, or “affected”. An element of the pathway is included or excluded in the resulting map if it matches with the selected filter criteria or not. The “affected” state is used to flag elements that may not be present given the user's selections, e.g., in a specific cell type a complex may not form if one or more of its proteins are not present, to guide the analysis and the data interpretation and to point out gaps in current knowledge.

The *annotator* allows to incorporate any kind of experimental measurements that can be matched to the identifiers of an element. Measurements can be coupled with graphical hints so that when the pathway is converted to a graphical representation elements with experimental measurements will be colored accordingly.

Lastly, the *exporter* permits the generation of identifier (gene) lists from a BCML file, enabling their use with analysis tools such as Gene Set Enrichment Analysis (GSEA), Fisher's Exact Test. Additionally, the format can be converted in a form amenable for impact analysis through the SPIA R package. The conversion can take into account the discriminative selection applied to the elements of the pathway, permitting analyses tailored for the user's experimental designs.

The BCML software suite is free and open source software, released under the GNU Lesser General Public License (LGPL) version 2.1.
